# Dietary Supplements: Regulatory Challenges and Research Resources

**DOI:** 10.3390/nu10010041

**Published:** 2018-01-04

**Authors:** Johanna T. Dwyer, Paul M. Coates, Michael J. Smith

**Affiliations:** 1 Office of Dietary Supplements, National Institutes of Health, Bethesda, MD 20892-7517, USA; coatesp@od.nih.gov; 2 National Center for Natural Products Research, University of Mississippi, MS 38677, USA; mjsmith073@olemiss.edu or m.j.smith@westernsydney.edu.au; 3 National Institute of Complementary Medicine, Western Sydney University, Penrith, NSW 2751, Australia

**Keywords:** dietary supplements, food supplements, supplement science, scientific challenges, regulatory challenges, natural health product, complementary medicine, traditional medicines, National Institutes of Health, Office of Dietary Supplements

## Abstract

Many of the scientific and regulatory challenges that exist in research on the safety, quality and efficacy of dietary supplements are common to all countries as the marketplace for them becomes increasingly global. This article summarizes some of the challenges in supplement science and provides a case study of research at the Office of Dietary Supplements at the National Institutes of Health, USA, along with some resources it has developed that are available to all scientists. It includes examples of some of the regulatory challenges faced and some resources for those who wish to learn more about them.

## 1. Introduction

The fundamental challenge in any discussion about the regulation of dietary supplements is that there is no global consensus on how the category of products known variously as dietary supplements, natural health products (NHPs), complementary medicines or food supplements in different countries is defined. For example, a product considered to be a dietary supplement and regulated as a food in the USA, in another jurisdiction may be considered a food supplement or a therapeutic good (complementary medicine) or a therapeutic good (prescription medicine) or potentially even a controlled substance. The situation is even more complicated when countries like China or India that have an existing regulatory framework for traditional medicine or phytomedicine that includes crude botanicals are considered. To add further to the confusion, many regulatory frameworks are changing.

Another challenge is that while all regulatory scientists want to protect consumers from harm, ensure that consumers have the ability to make informed choices about the products they use, and do the right thing, the scientific challenges and regulatory systems that have arisen to deal with them vary greatly from country to country. Even in countries with similar cultures, legal systems, and levels of economic development, regulations applying to dietary supplements vary considerably. Some of these differences are explored below, using examples from Australia, Canada and the USA, all English-speaking countries with largely similar cultures and legal systems to illustrate this point. The discussion of other countries with similar legal systems such as the United Kingdom, New Zealand and South Africa or other nations in the Americas, Europe, Africa and Asia, often with different cultures, legal systems, and levels of economic development is left for others with greater expertise and experience.

A final challenge is that “dietary supplement” health products are often very emotive and polarizing topics, evoking a diverse range of opinions and viewpoints. While some observers may contend that these products should be considered in a similar fashion to conventional drugs and foods, others believe that a more tailored approach is necessary since there is often a traditional or historical evidence base and products often contain multiple ingredients. Increasingly, this situation has become even more complex because of the lucrative nature of the global dietary supplement sector, increased involvement of a growing industry sector producing them, and the introduction of many new and innovative products onto the market. A detailed discussion of the politics of the subject is outside the scope of this paper. However, it must be recognized that politics may play both a positive and negative role in shaping both regulatory frameworks and research agendas. Irrespective of the reader’s point of view, this context is important in any discussion of dietary supplement products.

### 1.1. Importance of Research on Dietary Supplements

Until relatively recently, there was limited scientific research on dietary supplements and so little was known about them [1]. However, the prevalence of supplement use has increased dramatically over the past 20 years [2], and they have become a matter of consumer interest [3,4]. At the same time, the application of state-of-the art scientific methods to explore issues involving dietary supplements has advanced rapidly. The other invited articles in this special issue illustrate progress in our understanding of supplement science as it applies to several nutrients, including vitamin D, iron, omega-3 fatty acids, and iodine. Progress on botanicals and other non-nutrient ingredients (e.g., glucosamine, methylsulfonylmethane (MSM), coenzyme Q10) has been more challenging [5]. There is no global consensus in terminology for the category of products known variously as dietary supplements, NHPs, and food supplements in different countries and while we recognize this limitation, for the purpose of this article the term dietary supplement will be used to refer to such products as nutritional supplements, herbal medicines and traditional medicines. This article summarizes some of the scientific challenges in supplement research and some resources that may be useful in studying them. Most of the scientific challenges in supplement science are ubiquitous and global, so it is vital for scientists to collaborate across nations to help meet them without duplicating effort. A case study is provided by the work of the NIH Office of Dietary Supplements (ODS) which has been pursuing this goal since 2000. Some freely available resources and tools that ODS has developed for advancing health-related scientific knowledge on supplements are presented. The supplement marketplace is increasingly international, making collaboration between regulators essential since national decisions have international implications. Since products are consumed world-wide, calls for global quality standards are emerging. The remainder of the article focuses on regulatory challenges involving dietary supplements, and perspectives on how the regulatory systems in a number of different countries deal with them. Key resources for learning more about these approaches are provided.

### 1.2. Areas of Scientific Consensus about Supplement Science

Although there is broad consensus on the need for advances in science to make progress, opinions vary on the best paths to take and on priority areas for consideration.

#### 1.2.1. Quality

The supply of ingredients used in supplements has outpaced the availability of methods and trained personnel to analyze them [6]. For example, in 1994, when the Dietary Supplement Health and Education Act (DSHEA) first became law in the USA, about 600 U.S. manufacturers of supplements were producing an estimated 4000 products. By 2000, more than 29,000 supplement products were on the US market but few documented analytical methods or reference materials (RM) were available for these products. This growth in the market has also been evident internationally. For example, there are anecdotal reports that over 100,000 product license applications have been approved in Canada since the Natural Health Products Regulations came into force in 2005. The need for improving quality continues today, since now there are estimated to be more than 85,000 supplement products in the US marketplace and concerns about ingredient misidentification, safety concerns, and quality assurance/control problems continue to be important for the industry and the public [7,8].

The first step in characterizing supplement products is generally identifying the ingredients [9]. Plant identification is a particular challenge. Even when easily identified whole plants or plant parts are used, unless the chain of custody is tight, and the exact manufacturing process is known and well characterized, the quality of extracts and blends such as those found in many botanical products is difficult to ascertain. Reliable analytical methods to characterize the bioactive components in supplements are helpful, but even for the nutrients in supplements, specific analytical chemistry methods must be often developed [10]. The bioactives in supplements differ from those in foods in their matrices in that the forms, combinations, and doses in which they are consumed, and the circumstances under which they are used are likely to differ. Analytical techniques for other bioactives in supplements are further complicated because the active compound(s) are often unknown, and even when they are known, validated analytical methods may not exist for determining their content. Reference materials are often unavailable to compare results between different laboratories for research purposes and to monitor data and supplement quality.

#### 1.2.2. Safety

Manufacturers are prohibited from marketing supplement products that are unsafe or contain unsafe ingredients. This includes assuring that safe upper levels of intake for nutrients or maximum dosages for other constituents are not exceeded and ensuring that toxic contaminants are absent. Improved accuracy and precision of the nutrient measurements, bioactive marker compounds for other ingredients, natural toxins, toxic elements and/or pesticides in dietary supplement ingredients and finished products will be helpful to regulatory agencies.

#### 1.2.3. Efficacy

Demonstration of efficacy typically depends on a number of research approaches ranging from basic in-vitro research on the mechanisms of action to animal and human studies. For example, in the past, large and expensive clinical trials using poorly characterized herbal supplement products for which the mechanisms of action were not understood were performed, leading to results that were inconclusive and irreproducible [11,12,13]. These experiences led publishers and funders to demand better product characterization and funders to demand more mechanistic evidence of bioactivity. Once mechanistic plausibility is established, animal and small phase 1 and phase 2 trials should precede the launch of large phase 3 studies of efficacy. More and better clinical studies of the safety and efficacy of dietary supplements on “hard” health outcomes are also sorely needed. Health outcomes such as changes in validated surrogate markers for performance, functions, morbidity, and mortality from diseases or conditions are required rather than changes in biochemical measures in blood with unvalidated surrogate markers. The question of the use of evidence from traditional forms of health and healing such as Traditional Chinese Medicine (TCM) makes the question of efficacy often more complex. This is briefly explored in the regulatory section below.

#### 1.2.4. Translation of the Science

Widespread consensus exists on the need to translate the scientific evidence on supplements into appropriate recommendations, regulations, and policies that ensure the public health. Population-based prevalence estimates of supplement use are needed to estimate total exposures to nutrients or other bioactives that can be related to health outcomes [14]. Monitoring is especially important when supplementation is used as a public health strategy to fill nutrient gaps in deficient populations. It is also needed in other countries such as the USA where use of certain supplements is high, and where substantial proportions of total intakes of nutrients such as vitamin D and calcium come from supplements, especially among older adults [15].

## 2. Challenges and Resources: Regulatory Perspectives

As with other categories of regulated goods such as foods and drugs, the development of regulations is a balancing act where many different factors need to be taken into account. Notable among these are ensuring that products are of high quality and safe, that any claims made are truthful and not misleading, and that there is reasonable and appropriate access to the marketplace. All regulatory scientists want to both protect consumers from harm and support them in making informed choices about the products they include—or as importantly do not include—in their healthcare options. Appropriate regulatory oversight of this category is very challenging, and requires that scientists and regulators work together, as the former director general of the World Health Organization, Margaret Chan, MD urged [16]. This section provides a concise overview of how these regulations have been developed, and common themes as well as challenges faced in a global market.

### 2.1. Definition of “Dietary Supplements”

Although the definition of dietary supplement within a specific jurisdiction such as the USA is quite precise [17,18], a fundamental challenge to any discussion on regulation is that there is no global consensus on either what falls within this category or even what the category is called. Intuitively many equate a dietary supplement in the USA with a NHP in Canada or a traditional herbal medicine in the European Union or a complementary medicine in Australia, but this is not the case. For example, while melatonin is regulated in the USA as a dietary supplement and in Canada as a NHP, in Australia it is considered as a prescription medicine [19,20,21]. Dehydroepiandrosterone (DHEA) is readily available as a dietary supplement in the US, while in many other jurisdictions it is regulated as a controlled substance and is subject to significant regulatory oversight [22].

This situation is even more complicated when one considers that in addition to dietary supplements such as vitamins and minerals, many of these products come from traditional systems of health and healing such as TCM in China and Ayurvedic/Unani/Siddha medicine in India. For this reason, we must differentiate between the manner in which nations regulate the practice of medicine and the manner in which they regulate marketed products used in medical practice or as foods. In the U.S., the practice of medicine is regulated by the states, while marketed food and drug products in interstate commerce are regulated by the Federal government. Approaches and regulatory frameworks in many parts of the world, notably in Asia, reflect this fact with terminology and categories developed accordingly [23].

To assist in development of its Traditional Medicine Strategy 2014–2023, the World Health Organization refers to this category as Traditional and Complementary Medicines (T & CM) [16]. Although this classification does have significant limitations, it recognizes the fact that definitions for this category vary significantly globally. Descriptions of specific national/regional definitions and categories can be found through the list of resources in Table 1.

While it would be easy just to consider that the substance itself is the defining factor in determining whether or not a product is a dietary supplement, this is not the case. Two other important factors considered are the claim that the product is making and how the product is supplied or recommended (intended use). In many jurisdictions such as the USA, Canada and Australia, dietary supplements are considered suitable for self-selection without the need for the intervention of a practitioner or prescription. Here the claims that can be made are limited to minor conditions and to the support of health and wellness depending on the jurisdiction [24,25]. In other jurisdictions, notably those where a traditional form of health and healing is recognized, traditional and complementary medicine products are often prescribed, and in some cases supply is limited only to trained practitioners.

### 2.2. Regulatory Models

As with the definition of the products themselves, there is no consistent global approach to regulation, with many different frameworks developed that largely reflecting national and regional priorities and needs. That being said, there are a number of common themes and approaches that have been taken internationally.

#### 2.2.1. Where Does the Category Fall within Existing Legislation?

With a few exceptions, notably where traditional forms of health and healing exist, most countries do not regulate dietary supplements as a stand-alone category. Rather, they include them as a subset of existing legislation [17,18]. That is, they “hang from the hook” that is set in existing legislation. In the past, this was largely a question of whether these products should be considered a subset of drugs or foods; increasingly though, a third option is to capture them under existing regulations for biologics. It is important to note that overarching legislation is often one of the most important factors impacting the type of claim that can be made and what level of scrutiny and oversight will exist. For example, countries that regulate these products as a subset of drugs or therapeutic goods such as Australia, Canada and the European Union (EU) for traditional herbal medicines allow far more specific clinical claims to be made than in a jurisdiction such as the USA, where dietary supplements are captured in regulations under the existing food legislation, with their advertising regulated by trade regulations [20,25,26].

#### 2.2.2. Should They Be Regulated as a Group?

As noted above in many jurisdictions dietary supplements are simply captured under the existing food or drug regulations and legislation with no specific consideration for these products, in some cases specific regulations have developed to reflect the category. In these cases, two different regulatory models have typically been adopted that reflect their domestic use, national priorities and public health needs. In many jurisdictions, the first model applies. Dietary supplements are simply captured under the existing food or drug regulations and legislation. In that model, a wide range of products (typically herbal medicines, traditional medicines and dietary or nutritional supplements) reside under an umbrella term such as dietary supplements in the USA, complementary medicines in Australia or NHP in Canada [20,24,25]. In the second model, specific regulations are developed to deal with these products. In this case, specific categories are developed with very structured regulatory frameworks for specific types of T&CMs. This is particularly the case in countries with a strong traditional form of health and healing such as Chinese proprietary medicines in China (TCM), Ayurvedic medicines in India and Kampo medicines in Japan [23].

Irrespective of the approach taken, it is rare that one set of regulations will encompass all products commonly considered to be dietary supplement-like. Typical examples of this are guidelines and legislation related to advertising that apply irrespective of whether or not a product is considered to be a dietary supplement.

#### 2.2.3. Common Elements of Regulatory Frameworks

As with other forms of regulations, independent and irrespective of the approach taken, frameworks that deal with dietary supplements may contain a number of common elements, in this case often specifically developed to reflect the challenges and nature of the products. These common elements include: process for approval of a product to be sold; provisions related to manufacture and Good Manufacturing Practices (GMPs); reporting of adverse events; controls on labeling related to indications, contraindications and warnings; and, where claims are permitted, the type and quality of supporting evidence required. Again, the number and nature of these elements applied are determined by the specific regulations in place.

#### 2.2.4. Risk-Based Approach

Operationally, the regulation of dietary supplements faces a number of issues and challenges not shared with conventional drugs or even food products. Notable amongst these are the sheer number of individual dietary supplements on the domestic markets, often numbering in the tens of thousands, and the fact that the sector contains many different types of products often posing very different risks that are grouped together often by the fact that they do not fit under any other regulatory regime. In particular, considerable challenges are posed especially by herbal and traditional medicine products that contain crude botanicals and a complex milieu of potentially active moieties, unlike conventional allopathic pharmaceuticals.

While a completely pre-market approach, where all products and manufacturing sites are ‘approved’ before the dietary supplement is marketed would be the optimal situation, given the challenges mentioned above, this is often impractical. This has led to the development of regulatory frameworks that increasingly blend elements looking at products and sites both before they come to market as well as once they are available to consumers, or post-market. This regulatory oversight is sometimes referred to as a “life-cycle” approach. Examples of post-market regulatory approaches (i.e., once the dietary supplement is on the market) include target audits where dietary supplements already on the market are analyzed for quality or manufacturers are requested to submit evidence they may hold that supports a specific claim. The determining factor on which approach is applied is largely determined by risk posed to the consumer. Since most dietary supplements when appropriately manufactured are considered to be inherently low risk, increasingly regulatory frameworks are increasingly focused more on post-market review than pre-market licensure.

Even in countries that are in many ways socially, economically and legally similar, different approaches to the definition and regulation of dietary supplement health products are evident although they contain some common elements. Illustrative examples of this are evident in the different regulatory frameworks in place in the United States, Australia and Canada.

In the United States, dietary supplements are regulated under the Dietary Supplements Health Education Act of 1994 (DSHEA) as a subset of foods and limited to those taken orally. This approach is primarily post-market in nature. However, it does contain pre-market elements. For example, manufacturers must hold evidence to support their claims and they cannot make specific disease treatment claims but only claims related to nutritional support (which includes physiological structure and function) [20]. All products must carry a disclaimer on the label stating that claims have not been reviewed by the US Food and Drug Administration (FDA). Provisions also include a post-market site audit process for manufacturing sites for Good Manufacturing Practice compliance and mandatory reporting of serious adverse effects by manufacturers. Companies must notify the Food and Drug Administration before marketing products with new dietary ingredients (NDI) [27]. There is at present no indication that DSHEA will be substantially changed or modified by Congress, in recent years the regulatory authority has given more attention to the notification and classification of NDIs as well as the importance of Good Manufacturing Practices (GMP) [20].

In Australia, although a small number of these products are captured by a food standard, most are regulated as therapeutic goods under the Australian Therapeutic Goods Act. Products are referred to as complementary medicines and are legally defined as being a listed therapeutic good or a registered therapeutic good. The legislation itself does not define these terms, but a comprehensive set of guidelines describes how they are considered. Most complementary medicines are listed medicines and are managed through an online portal called the Electronic Listing Facility (ELF). Permitted claims are limited to minor, self-limited considerations and those traditional forms of health and healing such as traditional Chinese medicine. Evidence for efficacy is assured through a random and targeted post-market audit system and new listable substances are evaluated pre-market. As with all registered therapeutic goods, registered complementary medicines are evaluated pre-market for safety, quality and efficacy. Manufacturers of either finished listed or registered complementary medicines must undergo an on-site audit to ensure GMP [28].

In 2014, complementary medicines were included within a comprehensive review of regulations for all therapeutic goods and medical devices to be conducted by an external expert panel [29]. The Commonwealth government accepted the majority of the recommendations from the panel and preliminary draft legislation was made public in September 2017. Although one of the recommendations was to keep complementary medicines as a distinct category, some significant changes are proposed, allowing mid-level claims through a new third regulatory route between the listed and registered therapeutic goods process as well as changes to how advertising is approved and compliance management [25,30].

In Canada, the majority of these dietary supplement products are referred to as natural health products (NHPs) and are considered a subset of drugs under a specific set of regulations—the Natural Health Products Regulations. Products must undergo a premarket assessment for safety, quality and efficacy. This is done in part through an online submission process with permissible claims supported by Health Canada monographs. Producers of NHPs who wish to make novel claims not supported through the monograph process must submit a full dossier of evidence for review. The products can make therapeutic claims, but their use is limited to self-care situations. While manufacturers are required to have a valid site license following approved GMP guidelines, no pre-market site audit is needed; the process being primarily paper based [24]. To address the growing number of NHPs sold in a food-like format, Health Canada has created a new category of food currently defined through regulatory policy called “supplemented foods”. The category does allow for some health claims, but they are limited reflecting the nature of the products [31].

Unlike Australia, Canada is proposing to take different approach and rather than keeping NHPs as a distinct category, will include them in a self-care health product category together with non-prescription medicines and cosmetics. The intent of this initiative is to support informed consumer choice through a more consistent regulatory approach to these product categories that is based on risk. Key questions being explored deal with topics including evidence needed to support claims, provisions ensuring safety and quality and introduction of cost recovery framework [32].

The overviews above are brief and concise with more detailed information on these country specific approaches to be found through the list of resources in Table 1.

#### 2.2.5. Competing Types of Evidence

While it is clear that high quality scientific evidence is always required to support the quality of a dietary supplement, from a regulatory perspective the same may not always be true with regard the type and nature of the evidence required to support a product claim. Given the nature of the dietary supplement sector and the fact that it often encompasses traditional medicines with a long history of use, the question faced by regulators is how to balance the need for robust scientific evidence with a respect for diverse forms of health and healing.

Globally, no consistent approach has been taken in answering this question. In some jurisdictions such as Canada and Australia, the approach has been to link the form of evidence, whether it be traditional or evidence based from scientific research, to the level and type of claim that can be made. In these cases, typically products based on traditional evidence making traditional health care claims are ‘approved’ according to pre-cleared and approved sources of information such as monographs or labeling standards. For products making higher level, clinical claims, in a way similar to that for conventional pharmaceuticals, companies must supply a full dossier with appropriate supporting evidence such as that from randomized controlled trials (RCTs) [24,28]. In many countries such as the United States with no pre-market approval framework system, claims that can be made are more limited [17,18]. In countries with long-established traditional forms of medicines such as in China, India, and Japan, specific regulatory frameworks have been developed for these types of products with the type of claim that can be made and the evidence required to reflect this approach [23].

As the dietary supplement sector matures and develops and the market for raw ingredients becomes more global, establishing a balance between evidence generated by scientific research and that coming from traditional forms of health and healing is becoming increasingly demanding. This will be discussed later.

### 2.3. Evolving Regulatory Landscape—Challenging Issues

International regulatory frameworks are still considered by many to be a new and novel sector, although many of them are now more than two decades old. They were developed to reflect a time when the sector and nature of the market, not to mention the needs and demands of the consumer, were very different. This has meant that some decisions made in the past around policies and regulatory decisions may need to be revisited. These include the need to evaluate evidence of the “grandfathering” of dietary supplements already on the market when new regulations were implemented, the need to ensure that approaches are sustainable through cost-recovery mechanisms and the more global nature of the market place. Table 1 provides links to some of the regulatory frameworks of different countries that provide insights into the ways issues are dealt with in them.

Some of the key issues that commonly arise are:

#### 2.3.1. Evaluating Evidence for Product Claims

As the market for dietary supplements has increased, so has the amount and diversity of scientific evidence and research to support, or not support, their use. This market is made more complex when there are conflicting evidence bases and conflicting ways for evaluating them. For example, how, or should, traditional evidence be evaluated within the framework of traditional healing theories or those of allopathic evidence based medicine; what should be done when evidence from traditional forms of health and healing are not supported by more conventional evaluation mechanisms such as randomized clinical trials; and how can consumers, often wanting to explore both conventional and traditional medicine, be supported in making informed choices about including, or not including, these products in their health care options.

The original concept of Evidence Based Medicine is based on three basic premises—individual clinical expertise, the best external evidence and patients’ values and expectations [33]. The challenge faced by the regulator is to ensure that these are in play and to support consumers in making informed choices that are often made in a self-care setting.

#### 2.3.2. Questions at the Regulatory Interface

It has never been easy to distinguish between a dietary supplement and other categories such as conventional foods, drugs and biologics. As all these sectors have evolved, this question of product classification has become even more complex. Two of the main questions at the regulatory interface are: what are the boundaries are between dietary supplements and conventional foods and between dietary supplements and over-the-counter drugs?.

As the popularity of dietary supplements available in a food-like format such as a pre-prepared drink or bar has increased, the line between what a consumer would understand to be a food as compared to a dietary supplement has become increasingly blurred. In essence, how does the regulator provide for appropriate regulatory oversight? This has been particularly challenging for those jurisdictions that consider these products as a sub-set of drugs with regulation and often legislation governing them that is very different from that for foods. In these cases, the regulatory frameworks are more specific to such dosage forms as capsules, tablets and tinctures. The challenge is one primarily of balance in providing a regulatory approach that is appropriate and not unnecessarily restrictive with the need to ensure that consumers are aware that these food-like dietary supplements that they are considering are not typical foods. This lack of clarity is also challenging for the private sector in determining what regulatory framework applies to a product, either food or drug, that they wish to develop and bring to market. In Canada, this concern required the government to create a new category called “supplemented foods” distinct from NHPs where products in a food like format are considered as a subset of foods and not as natural health products [31]. In other jurisdictions such as Australia, authority has been given to the respective regulators to deem something to be either a therapeutic good or a food based a specific set of criteria [34].

The challenge at the over-the-counter (OTC)/dietary supplement interface is even more pronounced. A number of herbal medicines with a long history of use within the conventional health care model, such as senna and cascara, are regulated in most countries as OTC drugs rather than dietary supplements. As described above, Health Canada is proposing to address this issue in part by considering both NHPs and OTC drugs within a single regulatory approach for self-care products [32].

#### 2.3.3. Working on the Global Stage

Although science and research may be global, regulations are still made primarily to reflect domestic needs and pressures. This poses a challenge regarding dietary supplements and dietary supplement ingredients that are now often sourced and/or manufactured outside of the country where they are sold. In spite of calls for regulatory harmonization, examples of true harmonization are limited to regions such as countries in the Association of South East Asian Nations (ASEAN) with the lack of a coherent and consistent regulatory approach prohibiting this globally [35]. Even if regulatory harmonization is not possible, regulatory cooperation is often a viable option, taking into account inputs from stakeholder groups such as industry and not just governments. For example, to support cooperation between regulators, in 2005 in Ottawa, the World Health Organization supported the creation of the International Cooperation on Herbal Medicine (IRCH). IRCH now has over twenty members and provides a forum and mechanism for regulators to share information on safety issues and common challenges they all face [36]. Increasingly governments are working together as well as with other stakeholders such as industry and consumers to address common problems and in some cases to provide regulatory decisions in one jurisdiction that can be used as a basis for action in another.

#### 2.3.4. Strengthening Product Quality

As the dietary supplement market has become more global and lucrative, so have the importance of ensuring product quality and the challenges in doing so. There are increasing numbers of cases of adverse reactions and some fatalities due to contaminants or adulterants in the product rather than in the dietary supplement ingredients themselves. In some cases this has been due to intentional fraud by producers of these poor quality products who have developed sophisticated methods for overcoming existing regulations and oversight. This situation is explored in greater depth elsewhere in this paper.

### 2.4. Need for Continued Science in Support of Regulation

Irrespective of whether the goal is to support production of high quality products or to develop, apply or modify methods for evaluation of evidence in support of claims, the need for robust and relevant science and research on dietary supplements has never been more necessary. As regulatory frameworks evolve, many of the questions posed above will need to be addressed, balancing the need for robust science with a respect for traditional forms of health and healing.

## 3. Challenges: Scientific Perspectives

### 3.1. Issues Involving Human Requirements

Scientists often disagree about definitions of human requirements for bioactives and the implications for supplements. They differ on whether some non-nutrient bioactives are required for certain population subgroups and also on the health effects associated with the use of non-nutrient bioactives. It has been known for over 100 years that inborn errors of nutrient metabolism exist that can be remediated by supplying the lacking nutrient that has become conditionally essential. However, it is not clear that such a model based on single gene defects is useful for the amelioration of multigenic complex diseases. It is unclear that there are large numbers of individuals with common diseases and conditions such as type 2 diabetes or depression whose unique genetic characteristics cause them to have special nutritional requirements requiring supplements or medical foods [37].

Discoveries of genetic polymorphisms and the advent of inexpensive genetic tests that are widely available to consumers have nutritional implications. They have led to the rise of personalized or “precision nutrition” [38] and to the proliferation of boutique “personalized” eating plans and “precision” dietary supplements supposedly tailored to an individual’s genetic profile. The extent to which such supplements are efficacious in reducing chronic degenerative disease remains to be determined.

### 3.2. Supplement Quality, Safety and Efficacy

Challenges remain on the appropriate means for assuring supplement quality, safety and efficacy.

#### 3.2.1. Quality

Regulators, health professionals and manufacturers often disagree on how much quality testing is necessary for supplements. This is echoed by the World Health Organization’s Strategy on Traditional Medicines 2014–2023 [39] where quality is seen as a cornerstone of the sector. Botanical extracts and blends present particular challenges for detecting misidentification and contamination. The presence of adulterants and contaminants of both a biological and chemical nature in supplements is also challenging. Certain categories of supplements, such as athletic performance, sexual performance, and weight loss products, are particularly prone to the deliberate “spiking” with unlabeled extraneous or synthetic substances to confuse analytical techniques and even occasionally the addition of active synthetic drugs. Purity is a special problem for individuals with inborn errors of metabolism for specific nutrients such as vitamin B-6 or choline who require reliable, high quality sources of the nutrient. In countries that do not require that added nutrients be pharmaceutical grade or provide nutrients free to such patients, afflicted individuals must buy products that vary greatly in their quality on the open market.

The scientific challenges involved in all of the problems cited above depend in part on the adequacy and application of analytical methods. Analytical methods and reference standards are lacking for many of the thousands of different bioactive ingredients in dietary supplements. There is still disagreement about whether only a single officially endorsed method of analysis is acceptable. Any analytical method that is appropriately calibrated to a recognized reference standard should suffice but the onus is on the user of the method to demonstrate that affirmative requirements are met and that the method is suitable for its intended use and yields results that are accurate and precise. Methods that are suitable for foods may not be so for dietary supplements. Opinions also differ on whether government or the private sector is responsible for developing reference standards and analytical methods, and, if the private sector develops them, how they can be both kept independent and objective and made publicly available to avoid duplication of effort while preserving the marketing advantage of the developer. Tension also exists between researchers who desire ever more precise analytical methods for ingredients in dietary supplements and manufacturers who are concerned about the expertise and monetary costs required to apply some of the methods. A balance needs to be struck between the two.

#### 3.2.2. Safety

Apart from concerns related to product quality, the safety of dietary supplements depends largely on dose. High doses of some nutrients are more likely to pose problems than others, although there is disagreement about the levels at which problems arise. For example, some dialysis patients who are receiving very large doses of calcium and the active form of vitamin D on a chronic basis may exceed the Tolerable Upper Level (UL) and incur adverse effects on health, including calcification of the soft tissues [40]. Very high doses of vitamin D may also cause adverse effects in people with normal kidney function [41]. There is little evidence that usual doses and forms of these nutrients give rise to health problems [42]. The possibilities of excessive intakes of nutrients from dietary supplements are greater in countries with programs to fortify their food supplies than in others, and therefore they must also be evaluated [43,44,45,46].

Dose-response data for establishing safe levels of intakes of non-nutrient bioactives in supplements is frequently lacking [47,48]. Some dietary supplements containing non-target herbs added intentionally (like germander as an adulterant for skullcap), or others such as black cohosh, kava extract, green tea and others have been associated with liver injures of various types even after taking into account concomitant use with acetaminophen and alcohol and consumption while fasting [49]. Extracts that are used in bodybuilding and weight loss have also been linked to liver injury. This has led to studies of the composition of different supplements [50,51]. Causes of liver toxicity from supplements appear to be due to insufficient regulatory authority, inaccurate product labeling, adulterants and inconsistent sourcing of ingredients [52]. There is controversy about whether evidence of causality is sufficient for regulators to take action against supplements that seem to pose a hepatotoxic risk [53]. Some possible actions include requirements for warning labels with usage instructions as is done for drugs, or/and removal of products from the market. Adulterated or fraudulent tainted products sold as dietary supplements are already illegal and subject to recall [54].

Interactions of some ingredients in supplements with other dietary supplements, nutrients, prescription or over-the-counter drugs are well documented. Of particular concern are adverse reactions occurring with commonly used medications, such as anti-hypertensive and cardiovascular preparations [55]. In addition, much interest focuses around concomitant use of herbal medicines such as St. John’s Wort which has been shown to alter drug metabolism of a number of drugs notably those used in the treatment of HIV/AIDS, warfarin, insulin, aspirin and digoxin [56].

#### 3.2.3. Efficacy

Among the most hotly debated issues in supplement research is the type and amount of evidence needed to demonstrate the efficacy of dietary supplements. Many of the issues involving efficacy include those common in testing of all medications such as study designs, significance testing, appropriate outcomes, effect sizes, acceptable biomarkers of effect, and the differences between statistical and clinical significance. In order to be efficacious, dietary supplements must be bioavailable, and yet in some countries regulations do not require testing of supplements for disintegration and dissolution and some products on the market fail such tests. This is a matter of concern both to researchers and regulators since such results have a negative impact on studies of dietary supplement efficacy. In-vitro methods are available for testing disintegration and dissolution of drugs, and these are adaptable for use with dietary supplement products. Regulators in some countries insist on changes in health outcomes or in validated surrogate biochemical markers of effect on the causal pathway to a health or performance outcome. Others accept changes in intermediary biochemical markers that may or may not be surrogates of health outcomes. These considerations have come to the fore because supplements on the market in some countries apparently have little or no demonstrated efficacy. For example, one recent review of 63 randomized, placebo-controlled clinical trials of dietary supplements in Western adults found that in 45 of them no benefits were found, 10 showed a trend toward harm and 2 showed a trend toward benefit, while 4 reported actual harm, and 2 both harms and benefits; only vitamin D and omega 3 fatty acids had strong enough benefits and lack of harm to suggest possible efficacy [3]. This is an area of controversy that is highly polarized with questions being raised that depend on the type of dietary supplement being used, notably herbal medicines, the quality of the studies included in the review, and additional factors such as product quality of the supplement being evaluated that need to be taken into account [57].

#### 3.2.4. Standards of Efficacy for Traditional Natural Products

The traditional use of Chinese medicines, Ayurvedic medicines and other remedies is embedded in larger healing systems and cultural or metaphysical beliefs that are part of users’ larger and more holistic world views. Should usual standards for efficacy should apply to them when they are used in the traditional manner? Clearly such uses are quite different than the use of a single product or ingredient at much higher traditional doses and without such a cultural context.

### 3.3. Policy

Although policy issues arise with all types of dietary supplements, the examples below will focus on nutrient-containing dietary supplements since these are particularly germane to discussions of nutritional status.

#### 3.3.1. Nutrient Supplements Are Only One of Many Strategies for Improving Nutrient Intakes

There are many strategies for filling nutrient gaps in dietary intakes. They include nutrition education on appropriate food choices, fortification and enrichment that add nutrients to staple foods, genetic engineering that increases the nutrient content of a commodity itself either by genetic engineering/biotechnology, biofortification involving conventional breeding, and the use of nutrient containing dietary supplements. Dietary supplements provide concentrated sources of bioactives that are low or lacking in some individuals’ ordinary dietary intakes. The supplements can be used selectively by those whose diets have gaps in them. However, supplements have disadvantages. Their use depends upon individual motivations. Because they provide concentrated sources of bioactives at relatively high levels, they may increase the risks that some individuals will ingest excessive quantities and suffer health risks. Moreover, since dietary supplements can contain ingredients that lack a history of safe use, their long-term health effects may be unknown. The advantages and disadvantages of dietary supplements as a strategy to improve dietary intakes therefore must be carefully considered.

#### 3.3.2. Supplementation as a Strategy to Achieve Nutritional Adequacy

The cost-effectiveness of using supplements to fill gaps in nutrient intakes as opposed to other means such as fortification or nutrition education varies from one nutrient to another and by country, and so each situation is unique and must be evaluated independently. There are also questions about what the supplement should be, if supplementation is chosen. In countries where nutrient containing dietary supplements are common, the use of multivitamin-multi-mineral (MVM) supplements is often associated with a greater proportion of the population reaching the estimated average requirement (EAR) for nutrients [58]. However, for some of these nutrients, intakes are already adequate, so that the increased intakes may do little good, and in some cases supplements may increase the risk of exceeding the upper safe level (UL) of intakes.

#### 3.3.3. Monitoring of Supplement Use

Monitoring of supplement use is particularly important in countries where premarket approval is not required to detect potential adverse reactions. Dietary indicators are known to be imprecise and estimates of usual intake are lacking for many nutrients [59]. Biochemical indicators of deficiency are often not well linked with adverse health outcomes, underscoring the need for more attention to be paid to the development of agreed on measures of deficiency and excess [60]. Recent work on key nutrient biomarkers is now available, facilitating the monitoring of high risk groups, such as pregnant women for folate status [61,62].

#### 3.3.4. Authoritative Recommendations for Dietary Supplements

Health and nutrition experts differ on whether it is appropriate to include recommendations for nutrient containing dietary supplements in national health promotion and disease prevention recommendations. Many countries opt to recommend that adequate nutrient intake for the general public be achieved solely from foods, and reserve recommendations of specific nutrient supplements for specific subgroups in the population. Others recommend only food alone with no recommendations for special populations.

#### 3.3.5. Inclusion of Dietary Supplements in Food Programs to Reduce Malnutrition

There is pressure by industry to include MVM or other dietary supplements in food programs. However, there is little evidence that the target groups are deficient in the ingredients in the supplements, nor has it been demonstrated that provision of a supplement leads to better health outcomes.

#### 3.3.6. Stimulating Innovation

The development of new and more highly bioavailable forms of the nutrients, timed release, dosage forms, novel bioactive constituents and the appropriate application of new technologies such as nanotechnology are all important, but some pose new scientific and regulatory challenges.

## 4. Case Study: Office of Dietary Supplements (ODS), National Institutes of Health (NIH), USA

This case study highlights some examples of dietary supplement research supported by or conducted at the ODS, and provides some research tools it has developed that may be useful resources for scientists both there and abroad.

### 4.1. Background

Since its establishment in 1995 as part of the implementation of the Dietary Supplement and Health Education Act [17,18] of 1994, the ODS is the lead federal agency devoted to the scientific exploration of dietary supplements. Its mission is to support, conduct and coordinate scientific research and provide intellectual leadership to strengthen the knowledge and understanding of dietary supplements in order to enhance the US population’s health and quality of life. ODS’s four goals are to: expand the scientific knowledge base on dietary supplements by stimulating and supporting a full range of biomedical research and by developing and contributing to collaborative initiatives, workshops, meetings and conferences; enhance the dietary supplement research workforce through training and career development; foster development and dissemination of research resources and tools to enhance the quality of dietary supplement research; and translate dietary supplement research findings into useful information for consumers, health professionals, researchers, and policymakers.

Several of its major initiatives that have expanded the scientific knowledge base on dietary supplements are described elsewhere in this special issue of NUTRIENTS. They include studies to clarify the implications for public health of omega-3 fatty acids [63], iodine [64], vitamin D [65], and iron [66].

### 4.2. Research Resources and Tools

This section provides the details on freely available research resources developed by ODS that are available for scientists to use to enhance the quality of dietary supplement research and meet public health priorities, with a focus on those that may be useful to scientists in other countries.

### 4.3. Analytical Methods for Dietary Supplements

The rigorous assessment of dietary supplement ingredients requires accurate, precise and reliable analytical methods and matching reference materials. The ODS Analytical Methods and Reference Materials program accelerates the creation and dissemination of validated methods and reference materials. It provides resources for characterization and verification of supplement product content that enhance the reliability and reproducibility of research using these products and supports product quality [67].

The genesis of the program was the paucity of publicly available methods for the analysis of supplement ingredients [68,69]. In 2000, the US dietary supplement community tended to use proprietary or compendial methods for quality control operations, and scientists and laboratories often kept their proprietary methods to themselves. Negative publicity about discrepancies between label claims and the results of product testing performed by third parties led to some unsuccessful efforts on the part of the industry to pay a laboratory to develop and validate methods through the Association of Official Analytical Chemists International (AOACI). The program was not successful for several reasons, including lack of expert technical guidance and conflicting sponsor priorities. However, this early effort led to a collaboration between trade associations, ODS, the AOACI, the United States Pharmacopoeia (USP), NSF International, and others in an attempt to establish standard methods for dietary supplement analysis. The ODS became involved because explicit wording in DSHEA required the Government to use “publicly available” analytical methods for enforcement actions involving dietary supplements. In response to the need for such publicly available methods and to support efforts to validate methods used in biomedical research on dietary supplement ingredients, ODS established the Analytical Methods and Reference Materials (AMRM) program in 2002.

ODS has been involved in sponsoring the creation of AOAC Official Methods of Analysis for dietary supplements and in the development and dissemination of numerous analytical methods and reference materials for 15 ingredients in dietary supplements in the USA, 32 botanical identification and documentation projects, and 45 studies determining contamination and adulterants. It has also helped to develop guidance on the validation of identity methods for botanical ingredients [70] and the conduct of single-laboratory validation studies for dietary supplements, Appendix K, AOAC Official Methods of Analysis, and provided guidance to evaluation of the literature on botanical supplements [71,72]. The portion of the ODS website includes a searchable database of analytical methods; these can be accessed at: https://ods.od.nih.gov/Research/AMRMProgramWebsite.aspx.

ODS also supports the Dietary Supplement Laboratory Quality Assurance Program in which participants measure concentrations of active and/or marker compounds and nutritional and toxic elements in practice and test materials. Exercises have included water and fat-soluble vitamins, nutritional and toxic elements, fatty acids, contaminants (e.g., aflatoxins, polyaromatic hydrocarbons (PAH’s)) and botanical markers (e.g., phytosterols and flavonoids).

### 4.4. Reference Materials

ODS supports the development of certified reference materials for dietary supplement ingredients with assigned values for concentrations of active and/or marker compounds, pesticides, and toxic metals to assist in the verification of product label claims and in quality control during the manufacturing process. A reference material is a material that is sufficiently homogeneous and stable with respect to one or more specified properties, which have been established to be fit for its intended use in a measurement process. A certified reference material (CRM) is a reference material characterized by a metrologically valid procedure for one or more specified properties, accompanied by a certificate that provides the value of the specified property, its associated uncertainty, and a statement of metrological traceability. Certified reference materials can be used for laboratory proficiency studies, methods development, method verification, and method validation studies. Calibration standards are the single chemical entities necessary for construction of calibration curves for quantitative analysis and for confirming analyte identity. Several processes are used to produce calibration standards. ODS provided funding to the U.S. Department of Commerce’s National Institute of Standards and Technology (NIST) for the development and distribution of calibration standard solutions and matrix standard reference materials (SRM^®^; a NIST-trademarked type of CRM). The materials fall into one of the following categories: (1) pure chemical entities or their mixtures, including many nutrients and other ingredients in dietary supplements for use in establishing analyte identity and for calibrating instruments; (2) natural matrix materials that represent the supply chain of a particular dietary supplement, e.g., biomass (ginkgo leaves and powder), processed botanical ingredient (ginkgo extract), finished product; (3) natural matrix materials that cover a range of analytes including nutritional compounds, botanical marker compounds, and compounds with known health concerns (heavy metals, pesticides, plant toxins); and (4) Clinical materials that can be used to assist clinical laboratories assess nutrient status or exposure, such as the measure of measure of vitamin D status commonly used around the world, serum 25-hydroxyvitamin D [73,74,75]. ODS is now expanding efforts to develop biomarkers of nutrient exposure and status in blood and other biological specimens in relation to chronic disease risk in individuals and populations. ODS has worked with NIST to produce and make available reference materials for calibration of various laboratory methods. Supplementary Table S1 shows NIST Standard Reference Materials (SRM^®^) now available. Supplementary Table S2 shows dietary supplement and nutritional assessment SRMs that are currently in progress.

### 4.5. Dietary Supplement Databases

Two databases have been developed by ODS that are described elsewhere in detail [76,77,78,79,80]. The goal of the Dietary Supplement Label Database (DSLD) is to include labels for virtually all dietary supplements sold in the USA. This provides all the information on the product label including composition, claims, and manufacturer contact information. It now contains over 72,000 dietary supplement labels, with new labels added at the rate of 1000 per month. Used together with food composition databases it is possible to estimate total daily intakes of nutrients and other bioactive ingredients from both foods and dietary supplements. A mobile version of DSLD is now available for use on smartphones to enhance consumer access to it [78,80]. It is primarily aimed at researchers and so contains information about products that are currently on the market, as well as those that have been removed from the market.

The Dietary Supplement Ingredient Database (DSID) provides analytically derived information on the amount of labeled ingredients of a representative sample of commonly used categories of supplement products sold in the USA, including adult, child and prenatal MVM supplements and omega-3 fatty acids. DSID is now being expanded to examine botanicals and other ingredients in supplements that are of public health interest, such as green tea products. Calculators included with the DSID permit a consumer to examine how closely the labeled contents of a nutrient in a product compare to chemical analyses of all products in the category [79].

### 4.6. Nutrition Research Methods and Review Methodology

Systematic reviews of dietary supplements require special techniques. ODS has sponsored a series of technical reports on the application of review methodology to the field of nutrition and dietary supplements [81,82,83,84,85,86]. Staff have also collaborated in performing systematic reviews with other groups [87,88].

### 4.7. Population-Based Monitoring of Dietary Supplement Use

In collaboration with the National Health and Nutrition Examination Survey (NHANES) of the National Center for Health Statistics, ODS investigates patterns of dietary supplement use using national and other large cohorts, and assesses supplements’ effects on total nutrient intakes. Several studies have focused on adults [89], children [90,91], and others in the population and their supplement use. Other studies have focused on the contributions to total intakes of nutrients made by dietary supplements. Investigators at ODS have been active in funding monitoring efforts on the links between intakes of folic acid and health [92]. They have devoted particular attention to blood levels of folic acid and dietary intake patterns that are associated with very low and very high intakes of the nutrient [93,94,95]. The survey methods used are well documented and they may be useful for those in other countries planning similar population-based surveys to consult [96].

The motivations for use of dietary supplements are also documented; they often differ from those specified in regulations. NHANES contains several items that are consumer tested and available for use in other surveys on motivations. Knowledge of motivations can improve understanding of how people use these products and may provide clues for encouraging appropriate supplement use.

### 4.8. Translation of Supplement Science for Health Professionals and the Public

ODS has produced and periodically updates a library of more than two dozen fact sheets on the ingredients in supplements such as vitamin D, magnesium, and special products such as MVM supplements and products marketed for weight loss. There is a detailed version for professionals that is complete with detailed references, as well as easy-to-read versions for consumers in both English and Spanish. ODS also works with the National Library of Medicine (NLM) to produce and update a Dietary Supplement Subset of NLM’s PubMed. The National Center for Complementary and Integrative Health (NCCIH) at NIH produces a series of fact sheets on many botanicals and other non-nutrient bioactives in supplements that are also useful. They can be accessed at https://www.nccih.nih.gov. ODS also hosts an intensive, free 3-day course on issues in dietary supplement research annually for researchers. Further information about these and other projects is accessible at: https://www.ods.od.nih.gov.

### 4.9. Other Resources

In order to foster the development of appropriate study methods for dietary supplement research, ODS sponsors workshops on the latest knowledge and emerging approaches to the study of dietary supplements. It also supports the development of cutting–edge approaches to elucidate the mechanisms of action of complex botanical dietary supplements. It co-funds the Centers for Advancing Research on Natural Products (CARBON) with the NCCIM, including its program to develop high content high throughput methods to rapidly generate hypotheses on active compounds and the cellular targets. These and other resources are announced as they become available on the ODS website.

### 4.10. Fostering Use of Systematic Evidence Reviews in Policy Making and Clinical Practice

ODS has strengthened the scientific framework for developing dietary recommendations by encouraging the incorporation of systematic reviews into the development of the DRI. It has sponsored 18 systematic reviews on topics related to dietary supplements. These include ephedra, B vitamins, MVM supplements, omega-3 fatty acids, soy, probiotics, and vitamin D. The ephedra systematic review was helpful to the US government in banning ephedra products from the US market. The systematic reviews of omega-3 fatty acids funded over a decade ago and more recent updates on their associations with cardiovascular disease and infant health outcomes have been useful for planning intervention programs as well as for regulatory purposes. Current AHRQ reviews are available on the AHRQ website (https://www.ahrq.gov).

## 5. Future Needs

Attitudes toward safety, efficacy, and values about what is important in food and life will be important in determining future needs involving supplement science in the countries we have discussed and perhaps elsewhere in the world. Safety is critical, and requires better chains of custody and product characterization that exists at present for these products, particularly those involving global markets. Efficacy, that is that the health promotion claims for the product are true and not misleading is also critical. Demonstrating efficacy requires clinical studies with well defined products and rigorous experimental designs, and the studies must be replicable. To that end, many publishers now require that submitted manuscripts comply with established guidelines for the reporting of clinical trial results (e.g., CONSORT guidelines), while funders require demonstration of product integrity by applicants [97,98]. Finally there are issues of personal choice and values, sometimes involving the efficacy of supplements as complementary and alternative therapies that are part of a larger philosophical or religious world views and systems. These must be accommodated without abandoning safety.

Both basic and more applied challenges will continue well into the future. Much remains to be learned about the effects of bioactive constituents such as flavonoids in foods and dietary supplements on health outcomes, as many recent papers in Nutrients and elsewhere indicate [99,100,101]. More and better biomarkers need to be developed and their associations with health outcomes clarified [102]. Supplements intended to enhance sports performance [103] botanicals used for disease treatment [104] and those ingredients thought to slow aging [105] all require identification of valid biomarkers of efficacy as well as of exposure. The role of supplements and the gut microbiome also must be explored for its associations with common diseases and conditions [106]. The associations between supplement ingredients and health outcomes in chronic degenerative disease must be clarified [47,105,107,108,109]. High risk groups need more attention Certain subgroups within the population such as athletes consume very high amounts of some supplements and it is important to monitor them to prevent adverse outcomes and study the effects, if any, on athletic performance [110]. Others use supplements in the hope that they will improve cognitive performance [103]. Those who practice polypharmacy with prescription, non-prescription drugs and dietary supplements represent another high-risk group, and interventions to limit the potential for adverse events are needed [111,112]. Collaborations among scientists in many countries are needed to drive supplement science forward.

Irrespective of the type of health product, high quality science is fundamental to the success of any regulatory framework. Assessments of the safety, quality and efficacy of nutrients and other bioactives are needed to provide the scientific information that regulators need [113]. As mentioned earlier, the nature and diversity of the sector means that regulators face a number of very specific challenges for these low risk products. These include evaluating traditional evidence, dealing with products that contain multiple bio-actives and addressing the growing challenges of ensuring product quality. It is critical that scientists and regulators work together and learn from each other in both identifying issues and developing ways in which they can be addressed. Although regulatory challenges must be met at the national level, there must be due regard paid to the fact that national regulatory decisions about supplements have global implications.

## 6. Conclusions

Science is vital in regulatory settings, and there is no reason that science and regulation should be incompatible [114]. The challenges in supplement science and its regulation provide new opportunities for scientists and regulators to work together both nationally and internationally, to learn from each other, and to cooperate and when appropriate harmonize approaches to improve the public health.

## Figures and Tables

**Table 1:** Useful Global Resources on Dietary Supplement Regulatory Issues and Definitions.

Name	URL	Comments
USA		
FDA Food and Drug Administration Dietary Supplements	www.fda.gov/food/dietarysupplements/	Details on regulations, policies and guidelines dealing with dietary supplements
Australia		
Therapeutic Goods Administration (TGA)	www.tga.gov.au/complementary-medicines	Details of existing complementary medicine regulations, policies and guidelines
Food Standards Australia and New Zealand	www.foodstandards.gov.au/Pages/default.aspx	Details on food standards, policies and guidelines.
Canada		
Health Canada	www.canada.ca/en/health-canada/services/drugs-health-products/natural-non-prescription.html	Details on the existing NHP regulations, policies and guidelines as well as work underway with regards to a comprehensive approach to self care products
	www.canada.ca/en/health-canada/services/food-nutrition/legislation-guidelines/guidance-documents/category-specific-guidance-temporary-marketing-authorization-supplemented-food.html	Information on supplemented food category.
EU European Union		
EU Parliament and Council	ec.europa.eu/health/human-use/herbal-medicines_en	Details on the traditional herbal medicine directive re: member states.
European Food Safety Authority (EFSA)	www.efsa.europa.eu	Provides details and links to regulation of foods and food supplements.
China		
China Food and Drugs Administration (CFDA)	eng.sfda.gov.cn/WS03/CL0755/	Information on health food regulations including ‘blue hat’ process. (Note: English translation was not available).
China—Special Administrative Region of Hong Kong		
Health Ministry—Chinese Medicine Division	www.cmd.gov.hk/html/eng/important_info/regulation.html	Information on policies and regulation related to Chinese proprietary medicines.
Japan	www.mhlw.go.jp/english/topics/foodsafety/fhc/02.html	
Singapore		
Health Sciences Authority	www.hsa.gov.sg/content/hsa/en.html	Information on policies, regulation and guidelines related to health products and Chinese proprietary medicines. As a member state, resource to access work on regulatory harmonization of products within Association of South East Asian Nations (ASEAN).
New Zealand		
Medsafe	www.medsafe.govt.nz/regulatory/DietarySupplements/Regulation.asp	Provides information related to regulation, policies and guidelines dealing with dietary supplements.
India		
Food Safety and Standards Authority of India (FSSAI)	fssai.gov.in/home	Government direction, standards and regulation of health supplements and nutraceuticals. New regulations published in November 2016 take effect in January 2018. Health supplements are intended to supplement the diet of healthy individuals over 5 year, and levels of nutrients should not exceed RDA amounts.
Ministry of Ayurveda, Yoga, Unani, Siddha and Homeopathy (AYUSH)	ayush.gov.in	Policies, guidelines and regulations dealing with Indian traditional medicines.
WHO World Health Organization	who.int/medicines/areas/traditional/en/	Provides links to on-going work by the WHO including the Traditional Medicine Strategy 2014-2023, the International Regulation on the Cooperation of Herbal Medicines and various technical guidelines.
World Self Medication Industry	www.wsmi.org	Industry association website providing details on international approaches to over-the-counter medicines including dietary supplements.
International Alliance of Dietary/Food Supplement Associations (IADSA)	www.iadsa.org	Industry association website providing details on international approaches to dietary supplements.

## Supplementary Materials

The following are available online at www.mdpi.com/2072-6643/10/1/41/s1, Tables S1 and S2 Table S1 Standard Reference Materials (SRM^®^) available from the National Institute of Science and Technology, US Department of Commerce. Table S2 Dietary supplement and nutritional assessment Standard Reference Materials (SRM^®^) currently under development at the National Institute of Science and Technology, US Department of Commerce (as of December 2016).
