# Supplementary material for: Dietary Supplements: Regulatory Challenges and Research Resources
Source: Nutrients. 2018 Jan 4;10(1):41. doi: 10.3390/nu10010041 (PMC5793269; doi:10.3390/nu10010041)
Supplement: Supplementary file 1 [file nutrients-10-00041-s001.pdf]

Supplementary Table S1 Standard Reference Materials (SRM®) available from the National Institute of Science and Technology, US Department of Commerce

NIST Standard Reference Materials for Dietary Supplements Currently Available		
**SRM Number**	**SRM Name**	**Analytes**
3274	Botanical oils containing omega-3 and Omega-6 fatty acids (flax, borage, evening Primrose, Perilla)	Fatty acids
3532	Calcium-containing solid oral dosage form	Calcium, vitamin D-3
3254	Camellia sinensis (green tea leaves)	Catechins, caffeine, theanine
3255	Camellia sinensis (greet tea) extract	Catechins, caffeine, theanine
3257	Catechins calibraton solution	Catechins
3275	Fish oils containing omega 3 and omega-6 Fatty acids	Fatty acids
3246	Ginkgo biloba (leaves)	Flavonoids, terpene, lactones (ginkgolides), toxic elements, DNA
3247	Gingko biloba extract	Flavonoids, terpene, lactones (ginkgolides), toxic elements,
3248	Ginkgo-containing tablets	Flavonoids, terpene, lactones (ginkgolides), toxic elements,
3256	Green tea-containing solid oral dosage form	Catechins, caffeine, theanine
3264	Hypericum perforatum L. (St John's Wort) methanol extract	Hypericin, hyperforin, toxic elements
3530	Iodized table salt (iodine)	Iodine
3279	Multivitamin/multielement tablets	Vitamins and carotenoids (15) ,elements (18)
3250	Serenoa repens (fruit)	Fatty acids, phtosterols, DNA

3251	Sernoa repens extract	Fatty acids, phytosterols, carotenoids, tocopherols
3254	Soy flour	Isoflavones, nutrients
3237	Soy protein concentrate	Isoflavones
3236	Soy portine isolate	Isoflavones
3238	Soy-containing solid oral dosage form	Isoflavnones
3278	Tocopherols in editble oils	Tocopherols, tocotrienols
2972a	25-hydrowxyvitamin D calibration solutions	25(OH)D2, 25(OH) D3, and 3-epi-25(OH) D3
972a	Vitamin D metabolites in frozen human sera	25(OH)D2, 25(OH) D3, and 3-epi-25(OH) D3
2378	Fatty acids in frozen human serum	Fatty acids
2873	Vitamin D metabolites in frozen human serum (high level)	25(OH)D2, 25(OH) D3, and 3-epi-25(OH) D3
3950	Vitamin B6 in frozen human serum	Pyridoxal 5′-phosphate, 4-pyridoxic acid

Supplementary Table S2 Dietary supplement and nutritional assessment Standard Reference Materials (SRM®) currently under development at the National Institute of Science and Technology, US Department of Commerce (as of December 2016)

SRM#	SRM Name	Analytes
3281	Cranberry (fruit)	Anthocyanidins, proyanidins, organic acids,nutrients, DNA
3283	Cranberry extract	Anthocyanidins, procyanidins, organic acids
3284	Cranberry-containing solid oral dosage form	Anthocyanidin, procyanidins
3285	Mixed berry-containing solid oral dosage form	Anthocyanidin, procyanidins
3286	Organic acids calibration solution	Organic acids
3287	Blueberry (fruit)	Anthocyanidins
3291	Bilberry extract	Anthocyanidins

3282	Low calorie cranberry juice cocktail	Anthocyanidins, procyanidins, organic acids, nturients
3235	Sy milk	Isoflavones, nutrient
3262	Hypericum perforatum L. (St John's Wort) Aerial parts	Hypericin, hyerforin, toxic elements, DNA
3232	Kelp powder (Thallus laminariae)	Iodine, organoarsenic species, vitamin K
3243	Polycyclic aromatic hydrocarbons in Mate tea	Plycyclic aromatic hydrocarbons
3299	Curcuma longa L. (turmeric) Rhizome	Cucuminoids, DNA
3300	Cucuma longa L. (Turmeric) extract	Curcuminoids, DNA
3381	Yohimbe bark	Yohimbe
3382	Yohimbe extract	Yohimbe
3383	Yohimbe-containing solid oral dosage form	Yohimbe
3389	Ginsenosides calibration solutions	Ginsenosides
3279	Chromium-containing dietary supplement	Chromium, other elements
8650	Pueraria montana var. lobata (Kudzu) Rhizome	Isoflavones, DNA
8651	Pueraria montana var. lobata (Kudzu) extract	Isoflavones
8652	Kudzu containing solid oral dosage form	Isoflavones
8665	Zingiber officinale (ginger) Rhizome	Arsenic, lead
8666	Zingiber officinale (ginger) extract	Gingerols, (pesticides)
For the Dietary Supplement Quality Assurance Program	Kelp powder (Thallus laminariae)	Cis and trans vitamin K 1
Not assigned	Basil	Cis and trans vitamin K 1
Not assigned	Chondroitin sulfate (porcine)	Chondroitin sulfate
Not assigned	Chondroitin sulfare sodium (bovine)	Chondroitin sulfate
Not assigned	Spirulina powder	Zinc, vitamins K, B1, B2
8661	Panax ginseng (Asian Ginseng)	Ginsenosides

	extract	
3263	Hypericum perforatum L. (St John's Wort) CO2 extract	Hypericin, hyperforin, toxic elements
3265	Hypericum perforatum L. (St John's Wort) Solid Oral Dosage form	Hypericine, hyperforin, toxic elements
8653	Trifoliium pratense (Red Clover) flowers	(Isoflavones) , DNA
8654	Trifolium pretense (Red Clover) extract	Isoflavones
8655	Red clover containing solid oral dosage form	Isoflavones
8656	Actaea racemosa (Black Cohosh) Rhizome	(Triterpene glycosides), DNA
8657	Actaea racemosa(Black cohosh) leaves	Triterpene flycosides
8658	Actaea racemosa (Black cohosh) Rhizome extract	Triterpene glycosides
8659	Black Cohosh-containing solid oral dosage form	Triterpene glycosides
8660	Panaz ginseng (Asian Ginseng) Rhizome	On hold
8662	Elutherococcus senticosus (Siberian Ginseng) Root	On hold
8663	Elutherococcus senticosus (Siberian Ginseng) Root extract	Eleutherosides
8664	Ginsend-containing solid oral dosage form	Ginsenosides and eleutherosides
1588c	Organics in fish oil	Fatty acids, organic contaminants (e.g. polychlorinated biphenyls, pesticides)
3294	Chinese Black Cohosh (leaves)	Triterpene glycosides
3293	Chinese Black Cohosh (Rhizome)	Triterpene glycosideg
